# Neuronal intermediate filament neurologic autoimmunity in a patient with Merkel cell carcinoma receiving pembrolizumab

**DOI:** 10.1007/s10072-026-09500-8

**Published:** 2026-10-23

**Authors:** Ali Hijazi, Saffet Guleryuz, Mark A Walshauser, Ghazala Hayat, Maithreyi Chappidi

**Affiliations:** 1 Department of Neurology, Saint Louis University Hospital, Saint Louis, MO USA; 2 Department of Hematology and Oncology, Rosalind Franklin University, Chicago , IL USA; 3 Cancer Care Specialists of Illinois, O’Fallon, IL USA

**Keywords:** Immune checkpoint inhibitor, Paraneoplastic, Neuronal intermediate filament antibody, Merkel cell carcinoma

## Abstract

**Background:**

Neuronal intermediate filament (NIF) autoimmunity is an uncommon paraneoplastic neurologic syndrome that has increasingly been recognized in association with immune checkpoint inhibitor (ICI) therapy. Merkel cell carcinoma is among the malignancies most frequently associated with NIF antibodies, although reported cases remain limited.

**Case Presentation:**

A 55-year-old female with stage IIIA Merkel cell carcinoma developed vertigo, gait instability, dysarthria, and persistent right hemifacial and cervical spasms after receiving second dose of pembrolizumab for recurrent disease. Brain MRI was unremarkable, while continuous electroencephalography demonstrated encephalopathy without a definitive electrocerebral correlate, raising suspicion for subcortical myoclonus. Cerebrospinal fluid (CSF) analysis revealed lymphocytic pleocytosis, elevated protein. CSF and positive NIF antibodies using cell-based assay. Pembrolizumab was discontinued, and the patient was treated with high-dose corticosteroids followed by monthly intravenous immunoglobulin, resulting in near complete resolution of her neurologic symptoms after 6 months.

**Conclusion:**

This case expands the growing spectrum of ICI-associated NIF paraneoplastic neurologic autoimmunity complicating Merkel cell carcinoma. To our knowledge, persistent hemifacial and cervical spasm has rarely been reported in this clinical setting. Early recognition of this syndrome, prompt antibody testing, discontinuation of ICI therapy, and initiation of therapy may improve neurological outcomes while informing further oncologic management.

## Introduction

Immune checkpoint inhibitors (ICIs) have revolutionized the field of Oncology and are increasingly used in the treatment of various malignancies [1]. However, ICIs can lead to immune-related adverse events (irAEs), with immune activation involving the nervous system in 1–3% of cases [2]. There is a growing number of clinically defined neurological irAEs some with associated neural antibodies that are detected in serum or cerebrospinal fluid (CSF) [3]. A growing yet less recognized neural antibody related to ICI paraneoplastic neurologic autoimmunity is Neuronal Intermediate Filament (NIF) antibody, which has been described to cause a plethora of neurological manifestations [4]. We present the case of a patient with Merkel cell carcinoma who developed a subacute neurological syndrome following therapy with Pembrolizumab and was found to be positive for NIF antibodies in her CSF.

## History

A 55-year-old woman with a history of hypertension initially presented for evaluation of a left inguinal mass that had grown over several weeks. Ultrasound-guided core needle biopsy was done and was positive for small cell carcinoma on pathology. She underwent left inguinal lymph node dissection with two lymph nodes diffusely positive for Merkel cell polyoma virus stain. Her final diagnosis was T0pN1bM0, stage IIIA Merkel cell carcinoma of the left groin with unknown primary. She received adjuvant radiotherapy (60 Gy in 30 fractions). Initial oncoprotein antibody levels for Merkel cell carcinoma were greater than 115,000 and dropped to a nadir of 7,500 following treatment, and she achieved remission on positron emission tomography computed tomography (PET/CT). Around a year later, her oncoprotein levels increased to 17,800 prompting a repeat PET/CT which showed peri-aortic lymph node involvement. Following this relapse, she received two cycles of Pembrolizumab, 3 weeks apart with evidence of lymph node regression on repeat imaging.

The day after her second Pembrolizumab infusion, she developed vertigo, double vision, nausea and vomiting which were treated symptomatically with intravenous fluids and anti-emetics at an emergency department. Her symptoms continued to progress over the next two weeks with prominent fatigue and gait imbalance prompting an admission for further evaluation. During this initial admission, a brain magnetic resonance imaging (MRI) with and without gadolinium was unrevealing, and she was started on Prednisone 60 mg daily for presumed adverse side effects from Pembrolizumab as the etiology of her symptoms. On day 17 following second pembrolizumab infusion, she reported muscle spasms in her right face, jaw, and neck prompting her go an emergency department for a third time, and a Neurology consultation was obtained. Neurologic examination including mental status, cranial nerves, motor, sensory, coordination and reflexes were all noted to be within normal limits. MRI of the brain and orbits with and without gadolinium and a CT angiogram of the head and neck were within normal limits. A routine EEG did not reveal any epileptiform discharges, and the right hemifacial spasms were documented as muscle artifact without any electrographic correlate. She was treated symptomatically with Baclofen 10 mg three times daily and Levetiracetam 500 mg BID. Despite therapy, hemifacial spasms, imbalance, visual changes, and slurred speech persisted, and she experienced multiple falls and started using a cane. She was admitted for a third time at a University Hospital setting almost 4 weeks after symptom onset. Initial Neurological evaluation during the third admission revealed a mildly confused female patient oriented to person, place and time. Her language was intact but had mild dysarthria. She had brief right hemifacial and neck spasms that waxed and waned. Cranial nerves II-XII were intact. Strength was 4/5 in her right arm and 5/5 in all other extremities, and sensation to light touch was intact. She had brisk reflexes at 3 + in the right arm and leg and 2 + reflexes in her left arm and leg, with notable pronator drift on the right, absent Hoffman sign and down going plantar responses bilaterally. Dysmetria was noted on finger-nose-finger testing on the right but not on the left. Gait assessment was deferred due to high fall risk. The differential diagnosis of this presentation included infectious encephalitis, brain metastasis, metabolic or drug-related myoclonus, autoimmune encephalitis, and neurological deterioration associated with tumor progression. A comprehensive workup was obtained outlined as follows.

Complete blood count was notable for leukocytosis with white blood cell count of 11.5 × 10^9^/L with neutrophil predominance (84.6%). Serum electrolytes were notable for hyperkalemia (potassium 5.2 mmol/L). Serum creatinine, sodium, calcium, magnesium, phosphorous, and troponin, were within reference ranges. A review of labs drawn on the previous admission revealed a normal Vitamin B12, Folic acid, creatine phosphokinase (CPK), and Thyroid Stimulating Hormone (TSH) levels. A lumbar puncture was obtained with an opening pressure of 22 cm H2O and 32 cc of CSF collected. CSF analysis revealed lymphocytic pleocytosis with 18 × 10^6^/L nucleated cells (96% lymphocytes), 22 × 10^6^/L red blood cells, elevated protein (82 mg/dL), and high-normal glucose (74 mg/dL), normal Angiotensin Converting Enzyme level (2.1 U/L), and absent xanthochromia. CSF cytology showed rare atypical cells in a background of mature lymphocytes. Given the initial symptomatology and CSF findings, the patient was started empirically on intravenous acyclovir at 10 mg/Kg every 8 h to empirically treat for viral encephalitis. Levetiracetam dose was further increased to 1,500 mg twice daily due to no improvement on the lower dose and significant distress from symptoms. A comprehensive meningoencephalitis panel, cryptococcal antigen and Herpes Simplex Virus 1/2 polymerase chain reaction CSF returned negative, and acyclovir was discontinued. Given the elevated protein in CSF, history of cancer, and recent ICI therapy, the clinical presentation was concerning for an autoimmune/paraneoplastic process involving the CNS, and the patient was treated empirically with intravenous methylprednisolone 1,000 mg daily for 3 doses, awaiting the results of additional CSF studies. A repeat brain MRI with and without gadolinium was unremarkable. Continuous EEG for 72 h revealed generalized slowing, right sided myogenic artifact over the orbitalis, frontalis and chest-wall region, without electrocerebral correlate and no epileptiform discharges. These findings were interpreted by the Epileptologist as suggestive of encephalopathy and possible subcortical myoclonus versus clinical-only focal-onset seizures. The encephalopathy improved following therapy with steroids. However, no significant improvement in the hemifacial spasms was observed. Autoimmune and paraneoplastic antibody panels (Mayo Clinic Laboratories) were obtained from both serum and CSF to test for the following antibodies: GAD-65, AMPA-R, CASPR2-IgG, GFAP IGA, AGNA-1, ANNA 1–3, PCA 1–2, PCA-Tr, Amphiphysin, CRMP-5, DPPX, GABA-B-R, IgLON5, LGI1-IGG, mGLUR1, Neurochondrin, NIF, NMDA-R, PCA-TR, PDE10A, Septin-7, TRIM46. All serum antibodies returned negative. CSF returned positive for NIF light and heavy chains using cell-based assay. Reflex Immunofluorescence Assay (IFA) titer for NIF antibodies was > 1:256, raising suspicion for a CNS autoimmune process. The remainder of listed antibodies in CSF were negative.

The patient was offered additional therapy with intravenous immunoglobulin (IVIG), but she declined. At that point, she was ambulating with a wheeled walker. She was discharged home on Prednisone 40 mg daily, Levetiracetam 1,500 mg twice daily and Diazepam 5 mg as needed. Pembrolizumab was discontinued, she received first dose of IVIG one week after discharge, and prednisone was tapered by one-half dose every two weeks over 10–12 weeks. Her symptoms gradually improved, with complete resolution of hemifacial spasms by 3 months following initiation of IVIG therapy and discontinuation of ICI, though she continued to have imbalance. Due to suspicion of cervical myelopathy, a cervical spine MRI was obtained showing C5-C7 spondylosis, moderate central canal stenosis and mild T2 signal hyperintensity within the C2-C4 cervical cord. Neurosurgical evaluation was pursued without any surgical intervention. To rule out a sensory ataxia, an EMG/NCS was performed showing irritative myopathic process and a chronic neuropathic process, and a myositis panel returned positive for U1-RNP antibodies. She completed a total of 6 cycles of IVIG at a dose of 0.5 g/Kg every 4 weeks, with near resolution of her imbalance at 6 months follow-up. Unfortunately, her Merkel cell tumor progressed, and she was started on cisplatin and etoposide without additional ICI therapy.

## Discussion

The patient developed subacute neurological symptoms following the second cycle of ICI therapy with pembrolizumab, which initially raised suspicion for ICI toxicity and was managed with empiric steroid therapy. However, the symptoms progressed leading to significant disability. She had partial response to high dose steroids in the acute phase but achieved significant improvement once ICI was held and received several doses of monthly IVIG infusions.

Paraneoplastic neurologic autoimmunity with NIF antibodies has been described before with different underlying malignancies with some manifesting following ICI therapy. The most comprehensive cohort was published by McKeon et al., including 41 patients between 1996 and 2019 [5]. The study categorized patient presentations into three syndromes: encephalopathy predominant, cerebellar ataxia predominant, or myelo-radiculo-neuropathies. Five patients were found to have Merkel cell carcinoma, with three receiving Pembrolizumab. Two of those patients developed encephalopathy and one developed ataxia. There was no report of hemifacial spasm or subcortical myoclonus in these patients or any of the others in the cohort. NIF antibody paraneoplastic neurological autoimmunity was also described in a 67-year-woman receiving avelumab for Merkel cell carcinoma and developed cerebellar gait instability and third cranial nerve palsy [6]. Myoclonus in association with NIF antibodies was recently described in a 54-year-old man with primary pancreatic adenocarcinoma with liver metastasis who presented with encephalopathy and myoclonic jerks. However, this patient was treated with conventional chemotherapy rather than ICI [7].

Beyond its clinical rarity, this case highlights the diagnostic and therapeutic challenges posed by ICI-associated paraneoplastic neurologic autoimmunity. Programmed cell death protein-1 blockade enhances antitumor immunity by restoring cytotoxic T-cell function but may simultaneously disrupt peripheral immune tolerance, facilitating the emergence or unmasking of autoreactive lymphocyte populations directed against shared tumor and neural antigens [1, 3]. Merkel cell carcinoma is a highly immunogenic malignancy owing to its association with Merkel cell polyomavirus and the generation of tumor-specific immune responses, making it particularly susceptible to both the therapeutic benefits and immune-mediated toxicities of ICIs [1, 3]. NIF antibodies are considered biomarkers of an underlying T-cell-mediated autoimmune process rather than directly pathogenic antibodies, underscoring the importance of recognizing these syndromes early and initiating prompt therapy while discontinuing the offending ICI when clinically appropriate [4, 5]. Our patient’s marked neurological improvement following pembrolizumab discontinuation, high-dose corticosteroids, and maintenance IVIG is consistent with prior reports demonstrating that early immunosuppressive therapy can improve neurological outcomes in ICI-associated NIF autoimmunity, although long-term oncologic management may be complicated by the need to discontinue ICIs and transition to alternative systemic therapies [1, 3, 5, 6].

## Conclusion

This patient expands the growing body of literature in the field of paraneoplastic neurologic autoimmunity particularly within the realm of NIF antibody positivity in Merkel cell carcinoma, for which only a handful of cases have been described. To the best of our knowledge, the symptoms characterized by hemifacial spasms / subcortical myoclonus have not been previously emphasized in Merkel cell carcinoma patients receiving ICIs. This further highlights the evolving nature of the field of autoimmune neurology in the landscape of expanding ICI use in the field of Oncology.
